# Remorse for discrimination: The role of group dominance in judging hate crimes against subordinate group members

**DOI:** 10.1111/bjso.70008

**Published:** 2025-09-02

**Authors:** Andrés Gvirtz, Patrick F. Kotzur, Andrew L. Stewart, Felicia Pratto

**Affiliations:** ^1^ King's Business School King's College London London UK; ^2^ Department of Psychology Durham University Durham UK; ^3^ Hiatt School of Psychology Clark University Worcester Massachusetts USA; ^4^ Department of Psychological Science University of Connecticut Storrs Connecticut USA

**Keywords:** apologies, crimes, discrimination, intergroup relations, punitive attitudes, remorse, social dominance theory

## Abstract

Power, especially in the court system, is a potent determinant of intergroup relationships. Blind justice being only an ideal, public opinion can influence whether harm to low power groups is considered criminal and should be prosecuted. Our experiments investigated the impact of social dominance orientation (SDO) on the perceived appropriateness of punishment for harm to subordinate group members by dominant group members. Further, we examined the moderating role of a remorseful apology. We argue that perpetrators who do not show remorse towards their less powerful victim might be judged less harshly by those scoring high in SDO. Apologizing for the harm indicates a desire for social cohesion, which should appeal more to those low on SDO. We tested our hypothesis across three potential hate crimes: a privacy violation against a gay man (Study 1, *N* = 87 US‐Americans), a shooting of an unarmed Black man (Study 2, *N* = 91 US‐Americans), and an assault against an innocent refugee (Study 3a, *N* = 179 and 3b, *N* = 157 Germans). In three of four studies, people who desired group dominance advocated harsher punishment of remorsefully apologizing perpetrators. Our research contributes to the understanding of punitive attitudes across group boundaries with far‐reaching societal implications.

On 22 September 2010, Rutgers University freshman Tyler Clementi jumped to his death off the George Washington Bridge, announcing his intention on Facebook (Friedman, 2010). Clementi's roommate, Dharun Ravi, had used a webcam to show Clementi's romantic interaction with another man to peers (Parker, 2012). Clementi subsequently committed suicide, presumably because his sexual orientation became public without his permission and Ravi was ultimately charged and convicted of invasion of privacy, bias intimidation—a form of hate crime—and other charges (Zernike, 2012). Clementi's suicide sparked a US‐wide debate on bullying of and suicides among gay people (Parker, 2012). Was Ravi the one in the wrong, and should he offer a remorseful apology for his actions? Many were concerned with what crime(s), if any, Ravi should be charged with and how severe his punishment should be if convicted.

Public opinion can influence what prosecutors choose to do when confronted with possible criminal situations, and political activism can also establish what actions are considered criminal (e.g. Boccaccini et al., 2008; Friedman, 2009). In fact, the 1990 U.S. federal hate crimes statistics law was a product of lobbying by lesbian and gay activists that the seldom‐prosecuted assaults against gay men be documented and prosecuted by law enforcement agencies (Hulse, 2009). Hence, understanding the psychology—motivations, attitudes and justifications—behind people's senses of what behaviour should be punished not only reveals important aspects of social psychology, but also may have far‐reaching societal implications.

Cases such as Ravi's transgressions against a person in a lower‐power group present a situation of particular interest to social dominance theory for a complex set of reasons. Social dominance theory notes that well‐off human societies are hierarchical in nature, with some societal groups at the top (enjoying the benefits of distributing social value, including resources and ‘rights’), and other groups at the bottom (Sidanius & Pratto, 1999). The theory further explains that group‐based hierarchies remain unequal due to the confluence of institutional discrimination and individual discrimination, both of which are aided by legitimizing myths for why certain groups are more deserving than others. One tool social dominance theory has introduced for understanding legitimizing ideologies and the roles people may desire or play in institutions is the individual difference called social dominance orientation (SDO; Pratto et al., 1994). Those higher on SDO are defined to support intergroup hierarchies and accept inequality across a variety of power‐group distinctions, including prejudice and legitimizing myths, have been found to do so in many countries (e.g. Pratto et al., 2013) and are more often found in high‐power than in low‐power groups (Lee et al., 2011).

Central to the present research, social dominance theory characterizes institutional, group, and individual acts of intimidation, harassment and violence by dominants against subordinates, whether legal or not, as a major form of social control of subordinates (Sidanius & Pratto, 1999). We consider Ravi's actions to be a prime example of such hierarchy‐enhancing social control practices. Hierarchy‐enhancing practices are more supported by people high than low on SDO (Green et al., 2009; Sidanius et al., 1994). Helping to stabilize dominance societies, institutions may also include hierarchy‐attenuating forces like public defenders, egalitarian procedures and laws protecting members of subordinated groups like equal opportunity and hate crimes laws (Green et al., 2009; Sidanius et al., 1994). Because the criminal justice system typically functions in a hierarchy‐enhancing manner (Sidanius et al., 1994; Sidanius & Pratto, 1999), people who favour group‐based dominance are expected to favour stricter punishments for alleged criminals (Green et al., 2009; Sidanius et al., 2006). Further, Green et al. (2009) demonstrated that people low on SDO are more lenient in punishment recommendations for subordinate offenders.

However, when an alleged perpetrator has engaged in what social dominance theory considers harm and violence to intimidate subordinates, this is sometimes approved of by legal authorities, and prosecuting such persons might be opposed by those higher on SDO. Adding more complexity, although people lower on SDO would unambiguously disapprove of intimidation of subordinates, studies have shown that they do not support punitive policies in general (Green et al., 2009; Pratto et al., 1998). Thus, in a somewhat ambiguous situation such as whether Ravi committed crimes against Clementi, people's attitudes towards punishment may not show a consistent relation with their SDO.

In both legal systems and in interpersonal relationships, there is an additional potential complexity relevant to the balance of power between parties, namely whether a transgressor has shown remorse and truly apologized for victimizing another. In apologies for instances in which a dominant group or its institutions abused a subordinated group, such as genocide, boarding schools, forced resettlement, colonialism, unethical medical procedures (Abbott, 2022; Clinton, 1997; Levey, 2024; Van Assche et al., 2021), it is understood that a remorseful true apology is a ritual that debases a more powerful party in an attempt to restore its moral standing and restore social cohesion (Páez, 2010). These social functions of apology help to disentangle how SDO will relate to recommended punishment for dominant‐on‐subordinate violations.

In particular, for a higher‐power group member to show remorse for harm done to a lower‐power group member, this signals respect and concern for the subordinate and a desire for social inclusion (Páez, 2010). None of those signals are among the social goals vis‐a‐vis subordinated groups that people high on SDO generally hold (e.g. Sidanius et al., 2013), so we would not expect a remorseful apology to elicit leniency from them. In contrast, social inclusion and broad respect and concern for persons do characterize low SDO people (Pratto et al., 1994), so we expect they would become more lenient towards a high‐power transgressor who showed genuine remorse. As stated above, in the absence of an apology, the desired punishment for acts of high‐on‐low power harm will not be clearly associated with SDO. The present set of experiments was designed to test these predictions.

We point out another complexity in theoretical reasoning. Although people may find forgiveness easier if a perpetrator expresses remorse (Gold & Weiner, 2000), to those higher on SDO, remorse from a dominant person who has harmed a subordinate person abases the dominating person and shrinks the status hierarchy. Thus, apologies for the abuse of a subordinate group member may displease people higher on SDO because it violates their fundamental motive of hierarchy maintenance. Supporting our theorizing, people scoring lower on SDO have been shown to provide and reward intergroup apologies more than those scoring higher on SDO (Hornsey & Wohl, 2017). Further, SDO has also been shown to correlate with opposition to hierarchy attenuating intergroup apologies (Karunaratne & Laham, 2019).

The present research adds to the literature by testing our hypotheses across four experimental studies. All studies were conducted at the height of public discussions about bias crimes against minorities. Study 1 (*N* = 87) presents the initial test of our hypotheses based on the real‐world events of Ravi introduced earlier. As such, we tested the prediction that a remorseful intergroup apology (vs. no remorseful apology) will moderate the effect of SDO on participants' perceived justified severity of punishment after a possible hate crime committed against a gay man in the United States. Study 2 (*N* = 91) tests the theoretical generalizability of Study 1 by examining our hypotheses in the context of a potential crime a White police officer committed against an unarmed Black person in the United States. Studies 1 and 2 were not pre‐registered. Finally, pre‐registered Studies 3a (*N* = 179) and 3b (*N* = 157) further extend the previous studies across instances of harm and participants' culture by testing our hypotheses in the context of a potential crime committed against a Syrian refugee in Germany, by either a private person (Study 3a) or by a police officer (Study 3b). In all studies, we control for participants' attitudes (prejudice) towards the outgroup in question. This is because SDO and negative outgroup attitudes are typically highly correlated (Whitley Jr., 1999), and we wanted to make sure that it is indeed preferences for hierarchies rather than prejudice against a specific group that drive our effects.

In sum, our experiments add to the understanding of the motivations, attitudes and justifications behind which people punish what behaviour under which conditions in the context of potential hate crimes (i.e. crimes by dominant group members against members of a vulnerable category, typically in a low power position in society), and thus reveal important aspects of the social psychology of apologies, social dominance theory, and legal and social psychology in general, with far‐reaching societal implications.

## STUDY 1: SUBORDINATE VICTIM AS A YOUNG GAY MAN

Study 1 tested our hypotheses based on the real‐world events of Ravi described above. As such, our main aim for this study was to test whether SDO moderated the effect of offering a remorseful apology on views on how severely the alleged perpetrator should be punished. At the time of data collection, it was not known whether any prosecutors would charge Ravi, nor with which crimes.

### Materials and methods

#### Data collection and participants

The study was conducted mere months after the Ravi incident, and thus, was a salient topic at the time of data collection, particularly among university students in the United States. Participants were *N* = 87 undergraduate students at a US university who participated in partial fulfilment of a course requirement using paper‐and‐pencil questionnaires. The analysed sample was 48% female, 77% white and 19 years old on average (SD = 1.16). None were excluded. The experiment was approved by the University of Connecticut Institutional Review Board, Nr. X02‐167. The sample size was determined by the class size.

#### Design

In all four experiments, participants first read about a scenario in which a person in a dominant group helped bring about the death of a person in a subordinated group. After initial measures concerning what crimes participants viewed as appropriate and recommended punishment, they then read a brief paragraph that either included a full apology in which the dominant group member signalled respect and concern for the subordinate by taking responsibility, expressing regret for their actions and the harm they caused and wishing for social cohesion (remorse condition), or in which the dominant group member did not signal respect and concern for the subordinate by deflecting responsibility and offering alternative justifications for their behaviour (no remorse condition).

#### Procedure and measures

Participants read a short description about the Clementi suicide and Ravi's role in Clementi's death (for further details on the scenario, see Web Appendix A: Survey Study 1: Appendix S1). Participants were then asked to play the role of a jury member and place a checkmark next to any crime for which they believed Ravi should be charged: No charge, Invasion of privacy, Cyberbullying, Hate crime, Manslaughter, Second degree murder and First degree murder. Each crime was accompanied by a detailed description of what it constitutes to make sure participants knew what each crime meant. Participants also rated how much time in prison Ravi should spend if convicted for each crime on a 5‐point Likert scale, labelled 1 (*1 month–2 years*), 2 (*3–5 years*), 3 (*5–7 years*), 4 (*8–10 years*) and 5 (*10 or more years*).

After this initial assessment, participants were randomly assigned to one of two conditions, which we fictionalized. In the Remorse condition, Ravi offered a remorseful apology for contributing to Clementi's death, signalling respect and concern for the victimized by taking responsibility, expressing regret for their actions and the harm they caused, and wishing for social cohesion. Participants in this condition read:

I'm sorry about your son. I had no idea my actions would want to make him take his own life. I feel so terrible. I didn't realize before that being gay could be so painful. I am learning a lot and I just hope that somehow, through this awful loss, we can learn to accept one another.

In the No Remorse condition, Ravi did not express remorse for contributing to Clementi's death. Rather, one might consider this non‐apology to *not* include signals of respect and concern for the subordinate by including a deflection of responsibility and offering alternative justifications for the behaviour (see Páez, 2010). Participants in this condition read:

I'm sorry about your son. I didn't know he was so fragile. It just seemed to me that if he didn't mind getting it on with a man, he wouldn't mind everyone knowing about it. I don't see why anyone thinks this is my fault. Either you're a man or you're not. I know what I am.

Participants then rated how severely a judge should punish Ravi on a scale from 1 (*very lenient*) to 7 (*very strict*). At the end of the study, participants completed an SDO scale, attitudes towards gays and lesbians scale, and basic demographic information. The 16‐item SDO scale (Pratto et al., 1994, alpha = .89) included items like ‘This country would be better off if we cared less about how equal all people were’, and is known to be stable over 3‐month period (e.g. Pratto & Shih, 2000). The 6‐item attitude towards gays and lesbians scale (Herek & Capitanio, 1995) included items like ‘Sex between two men is just plain wrong’.

### Results

#### Before the experimental manipulation

Table 1 lists, for each crime, the percentage of participants who believed Ravi should be charged, and the mean degree of punishment he should experience if convicted. Table 1 also lists the correlations between recommended charges and prison time with SDO. Overall, SDO neither significantly correlate with indicating which crimes Ravi should be charged, nor with the punishment Ravi should receive if convicted. Thus, before our experimental manipulation, SDO did not play a role in judging crimes against Tyler Clementi, a gay youth.

**TABLE 1:** Type of crime, percentage of participants who checked each crime and the correlations of these choices with social dominance orientation before the experimental manipulation, Study 1.

Type of crime	Percentage checked	Punishment	Correlations with SDO	
			Checked	Punishment
No crime	0%	–	–	–
Invasion of privacy	97%	1.55 (0.86)	−.06^ns^	−.04^ns^
Cyberbullying	86%	1.57 (0.82)	−.05^ns^	−.18^ns^
Hate crime	63%	2.59 (1.12)	−.10^ns^	−.07^ns^
Manslaughter	54%	3.51 (1.13)	−.04^ns^	.00^ns^
Second degree murder	0%	4.27 (1.02)	–	.09^ns^
First degree murder	0%	4.62 (1.05)	–	.16^ns^

*Note*: *p < .05, **p < .01 and ***p < .001. Punishment indicates how lenient (1) or strict (7) a judge should be on Ravi, with means presented with standard deviations in parentheses. *N* = 87.

#### After the experimental manipulation

We conducted a multiple regression analysis with condition, attitudes towards lesbians and gays, SDO and the interaction between condition and SDO predicting the severity of punishment that Ravi should experience if convicted. Table 2 displays the descriptive statistics, including correlations, among the variables used in the regression analysis. Consistent with previous research (Pratto et al., 1994), SDO correlated positively with negative attitudes towards lesbians and gays, and those attitudes correlated negatively with the severity of punishment recommended for Ravi. Hence, sexual orientation prejudice did play a role in whether participants perceived Clementi to warrant justice.

**TABLE 2:** Means, standard deviations and correlations among variables included in the regression analysis (*N* = 87), Study 1.

Variables	*M*	SD	2.	3.	4.
1. Condition	0.47	0.50	.11^ns^	.03^ns^	−.33**
2. SDO	2.76	0.95	–	.39***	−.00^ns^
3. Attitudes	2.56	1.45		–	−.32**
4. Punishment	5.60	1.20			–

*Note*: *p < .05, **p < .01 and ***p < .001. Condition was coded such that 1 = Remorse, 0 = No remorse response from Ravi. Attitudes = negative attitudes towards lesbians and gays. Punishment indicates how lenient (1) or strict (7) a judge should be with Ravi.

Abbreviation: SDO, social dominance orientation.

Table 3 displays the results of the regression analysis. Overall, the regression model was statistically significant, *F* (4, 82) = 7.85, *p* < .001, and explained 24% of the variance in the severity of punishment. We found statistically significant main effects of condition (1 = remorse, 0 = no remorse, *b* = −2.30, SE = 0.71, *p* = .002) and attitudes towards lesbians and gays (*b* = −0.33, SE = 0.09, *p* < .001) on the severity of punishment. People in the remorse condition and people with more prejudice recommended more lenient punishments.

**TABLE 3:** Multivariate regression analysis of punishment, Study 1.

Predictors	*b*	SE
0. Intercept	6.80***	0.46
1. Condition	−2.30**	0.71
2. SDO	0.01^ns^	0.16
3. Condition × SDO	0.53*	0.24
4. Attitudes	−0.33***	0.09
Observations	87	
*R* ^2^ [*R* ^2^_adj_]	27.7% [24.2%]	

*Note*: *p < .05, **p < .01 and ***p < .001. Condition was coded such that 1 = Remorse, 0 = No Remorse response from Ravi. Attitudes = attitudes towards lesbians and gays. Punishment indicates how lenient (1) or strict (7) a judge should be with Ravi.

Abbreviation: SDO, social dominance orientation.

**FIGURE 1:**
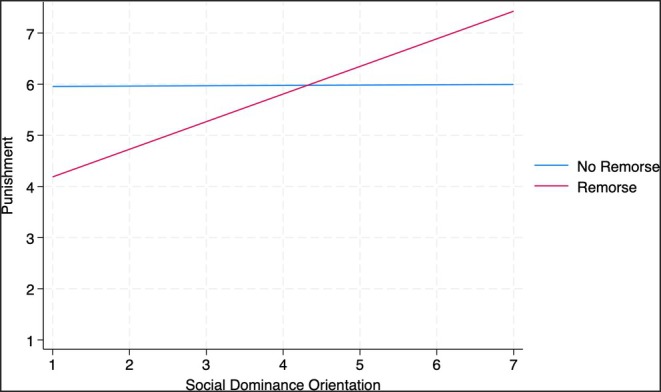
Interaction between social dominance orientation and the experimental manipulation predicting severity of punishment, Study 1 (Simple Slopes).

In addition, as predicted, we found an interaction between SDO and our experimental manipulation (see Figure 1, *b* = 0.53, SE = 0.24, *p* = .030). As can be seen by the simple slopes, when Ravi showed remorse for his actions (red line), people who scored higher on SDO recommended stricter punishment for Ravi (*b* = 0.54, SE = 0.19, *p* = .006), whereas people who were lower on SDO recommended less strict punishment for Ravi. This effect was not reliable in the no remorse condition (*b* = 0.01, SE = 0.16, *p* = .969).

### Discussion

Our main aim for Study 1 was to test whether offering a remorseful apology for a dominant‐on‐subordinate person attack moderated the effect of participants' SDO on how severely an alleged perpetrator should be punished. Echoing some prior research on individual legal apologies or intergroup apologies, the remorse condition reduced the severity of punishment strongly, but prejudice against the victim reduced the severity of punishment as well. As predicted, we uncovered an interaction effect, with participants scoring higher on SDO endorsing more severe punishments after the alleged perpetrator showed remorse in an apology compared to participants scoring lower on SDO. This suggests that signals regarding the meaning of actions for relations between dominants and subordinates—a remorseful apology—meaningfully qualify the envisioned punishment of the alleged perpetrator.

Although drawn from real events and consistent with our theorizing, Study 1 has limitations. First, given that student participants might have considered themselves to be peers of Ravi and Clementi, it is important to test our reasoning in populations that are not as close to the situation in the scenario. Second, Study 1 tested a particular, complex potential hate crime and pertained to a member of a sexual minority. Thus, the generalizability of our theorizing to other kinds of subordinated groups remained untested.

## STUDY 2: SUBORDINATE VICTIM OF VIOLENCE: BLACK MAN

Study 2 was designed to replicate the hypothesized effects and to remedy the two limitations of Study 1. As such, we tested our hypotheses based on a different set of newsworthy real‐world events—violence against unarmed members of racial minorities—using a more heterogeneous sample of US adults.

### Materials and methods

#### Data collection and sample

The study was conducted in close temporal proximity to several incidents of a similar nature as the stimuli. In particular, the police killing of 18‐year‐old Michael Brown sparked a larger debate around the frequent killings of unarmed Black people, and the subsequent exonerations of police officers in the United States (Harkinson, 2014). With our study taking place mere months after the event, such incidents were a salient topic at the time of data collection, particularly in the United States. *N* = 100 US adult participants were recruited through Amazon Mechanical Turk (MTurk) in exchange for a small monetary reward. Nine participants who did not complete the key measurements were excluded. The remaining sample of 91 participants was 44% female, 74% white and 37 years old on average (SD = 11.77). The experiment was approved by the Clark University Institutional Review Board, Nr. 2015‐N018A. The sample size was determined by the available funding.

#### Procedure and measures

Using the same procedure and design as Study 1, participants read a fictional short description about the death of a racial minority member—Anthony Taylor, a Black man—and White Officer Luke Clark's role in his death. In the scenario, modelled on real‐world events happening at the time of data collection (Whiting, 2022), Taylor was shot and killed by Clark, who wrongly assumed that Taylor was carrying a weapon (for further details on the scenario, see Web Appendix B: Survey Study 2: Appendix S1). Participants were then invited to place a checkmark next to the crimes with which they believed Officer Clark should be charged. These crimes were as follows: No charge, Hate crime, Involuntary manslaughter, Manslaughter, Second degree murder and First degree murder. Each crime was again accompanied by a detailed description of what constitutes the crime. Participants also rated how much time in prison Clark should spend if convicted for each crime on the same 5‐point Likert scale as in Study 1, labelled 1 (*1 month–2 years*), 2 (*3–5 years*), 3 (*5–7 years*), 4 (*8–10 years*) and 5 (*10 or more years*).

After this initial assessment, participants were again randomly assigned to one of two conditions. In the Remorse condition, Clark offered a remorseful apology for contributing to Taylor's death by signalling respect and concern, including taking responsibility, expressing regret for his actions and the harm they caused, and wishing for social cohesion. Participants in this condition read:

I'm sorry about your son. I felt so terrible when I realized that he didn't have a gun with him. I never intended to take his life. It was a split‐second decision, I thought my life was endangered. I feel deeply sorry about what happened and wished I could redo my mistake. I offer my sincere condolences.

In the No Remorse condition, Clark did not express remorse for contributing to Taylor's death, but rather deflected responsibility and offered alternative justifications for their behaviour:

I'm sorry about your son. If I would be back in the situation, I would have warned him louder, hoping that he didn't move so that I didn't have to assume he was pulling out a weapon. I can't really see why anyone thinks this is my fault. As an officer in the line of duty your life is threatened all the time, you have to make decisions.

Participants then rated how severely a judge should punish Clark on a scale from 1 (*very lenient*) to 7 (*very strict*). At the end of the study, participants completed a 16‐item SDO scale (Pratto et al., 1994, alpha = .95), a 20‐item attitude towards Blacks scale (Brigham, 1993, alpha = .94) and basic demographic information. The attitudes towards Blacks scale included items like ‘I would rather not have Blacks live in the same apartment building as I live in’.

### Results

#### Before the experimental manipulation

Table 4 lists, for each crime, the percentage of participants who believed Clark should be charged, and the degree of punishment he should experience if convicted. Table 4 also lists the correlations for recommending charging Clark with each crime and the prescribed length of prison time with SDO.

**TABLE 4:** Type of crime, percentage of participants who checked each crime and the correlations of these choices with social dominance orientation before the experimental manipulation, Study 2.

Type of crime	Percentage checked	Punishment	Correlations with SDO	
			Checked	Punishment
No crime	19%	–	.31**	
Hate crime	8%	3.23 (1.69)	−.16^ns^	−.29**
Involuntary manslaughter	27%	2.61 (1.27)	.05^ns^	−.26*
Voluntary manslaughter	18%	3.28 (1.31)	−.14^ns^	−.14^ns^
Second degree murder	43%	4.08 (1.15)	−.28**	−.20^ns^
First degree murder	1%	4.57 (1.12)	.14^ns^	−.25*

*Note*: *p < .05, **p < .01 and ***p < .001. Punishment indicates how lenient (1) or strict (7) a judge should be with Officer Clark, with means presented with standard deviations in parentheses. *N* = 91.

Overall, SDO did not significantly correlate with indicating which crimes Clark should be charged with, but it did correlate with the punishment Clark should receive if convicted. Higher SDO correlated with lower punishment. Thus, before our experimental manipulation, high SDO already implied a lower punishment for Officer Clark for causing the death of Anthony Tyler, a Black man.

#### After the experimental manipulation

As in Study 1, we conducted a multiple regression analysis with condition, negative attitudes towards Blacks, SDO and the interaction between condition and SDO predicting the severity of punishment that Clark should experience if convicted. Table 5 displays the descriptive statistics, including correlations, among the variables used in the regression analysis.

**TABLE 5:** Means, standard deviations and correlations among variables included in the regression analysis (*N* = 91), Study 2.

Variables	*M*	SD	2.	3.	4.
1. Condition	0.51	0.50	.15^ns^	.10^ns^	−.06^ns^
2. SDO	2.49	1.23	–	.80***	−.44***
3. Attitudes	2.60	1.15		–	−.41***
4. Punishment	4.63	1.87			–

*Note*: *p < .05, **p < .01 and ***p < .001. Condition was coded such that 1 = Remorse, 0 = No remorse response from Officer Clark. Attitudes = negative attitudes towards Blacks. Punishment indicates how lenient (1) or strict (7) a judge should be with Officer Clark.

Abbreviation: SDO, social dominance orientation.

We found SDO correlated positively with negative attitudes towards Blacks, and negative attitudes correlated negatively with severity of punishment recommended for Clark. However, the condition did not correlate with punishment, providing a first indication that the manipulation might not have been successful.

Table 6 displays the results of the regression analysis. Overall, the regression models were statistically significant, *F* (4, 86) = 6.27, *p* < .001, explaining 19.0% of the variance in severity of punishment. Condition (*b* = −1.08, SE = 0.81, *p* = .185), attitudes towards Blacks (*b* = −0.28, SE = 0.26, *p* = .284) as well as the interaction effect (*b* = 0.45, SE = 0.29, *p* = .131) were not significant predictors.

**TABLE 6:** Multivariate regression analysis of punishment, Study 2.

Predictors	*b*	SE
0. Intercept	7.04***	0.59
1. Condition	−1.08	0.81
2. SDO	−0.70*	0.27
3. Condition × SDO	0.45	0.29
4. Attitudes	−0.28	0.26
Observations	91	
*R* ^2^ [*R* ^2^_adj_]	22.6% [19.0%]	

*Note*: *p < .05, **p < .01 and ***p < .001. Condition was coded such that 1 = Remorse, 0 = No Remorse response from Officer Clark. Attitudes = negative attitudes towards Blacks. Punishment indicates how lenient (1) or strict (7) a judge should be with Officer Clark.

Abbreviation: SDO, social dominance orientation.

### Discussion

Our aims for Study 2 were (a) to test our hypotheses on a different set of fictionalized real‐world events than Study 1—an alleged crime by a White police officer against a Black minority member—(b) using a heterogeneous sample of US adults. Here, SDO was overall *negatively* related to the desire to punish the White officer, which contrasts with previous literature that finds SDO to be associated *positively* with punitive attitudes (e.g. Sidanius et al., 2006), but is consistent with social dominance theory's tenet that the police and military often serve to maintain group‐based dominance and thus appeal more to those higher on SDO (Sidanius & Pratto, 1999). One might recall that part of the national conversation around incidents involving White officers and unarmed Black civilians included a backlash in defence of police, exemplified by the ‘Blue Lives Matter’ movement. The effect of SDO correlating strongly negatively with prescribed punishment in Study 2 was more robust than the hypothesized condition main effect and moderating role of a remorseful apology. In other words, the hierarchy‐enhancing harm to a subordinate group member might have been enough to lead those endorsing hierarchies to trust the alleged perpetrator's actions and absolve him more than those who endorse equality. Previous work has found that scoring high in SDO is negatively associated with support for the Black Lives Matter movement (Azevedo et al., 2022).

There are multiple reasons why the manipulation might not have worked as anticipated. First, we cannot rule out that participants did not read the manipulation text with a sufficient level of care to be affected by it. A common problem in survey‐based research is that participants sometimes follow a satisficing rather than an optimizing strategy (Krosnick, 1991, 1999). Whereas optimizing participants meaningfully engage with the materials, satisficing participants ‘interpret each question only superficially and select what they believe *appears* to be a reasonable answer to each question without referring to any psychological cues specifically relevant to the attitude’ (Krosnick, 1991). Indeed, more recently, online data providers, like M‐Turk and Prolific, openly propagate the usage of attention checks to manage data quality on their platforms, something we did not do in Study 2, where 9% of our sample also skipped key questions and scales. As such, it might simply be that the manipulation did not work because participants did not engage with the manipulation and questionnaire sufficiently—a possibility we explore in Study 3. A further possibility is that, due to the slightly noisier design and non‐completion, Study 2 may have been underpowered to detect the hypothesized interaction reliably.

Another potential reason might be theoretical. The perpetrators in Study 1 and Study 2 have different levels of power and authority afforded to them. Whereas a police officer (like in Study 2) is equipped with substantial power (e.g. weapons, licence to give orders and interfere in other's privacy and lives), this is less the case for a private individual (like in Study 1). Thus, it may be the case that individuals acting on behalf of the state are consensually held to a higher standard when judging their actions, meaning that apologies containing remorse or not may weigh less for them than for private individuals. In such a case, there may be simply no main effect of displaying remorse that can be moderated by an observer's SDO, a possibility we explore in the next set of studies.

## STUDIES 3A AND 3B: SUBORDINATE VICTIM IS WAR REFUGEE

Pre‐registered Studies 3a and 3b were designed to address some of the questions raised by the previous studies. The primary goal of Studies 3a and 3b was to offer a potential explanation for why we found the hypothesized effects in Study 1, but not in Study 2. To do so, we tested whether the alleged perpetrator was a private individual or a representative of the state, which might account for the different findings. We additionally improved the design of our study by including attention checks to rule out that the discrepancy between findings was due to systematically different levels of participants' motivation and attention across studies.

What is more, we aimed to further our understanding of the generalizability of results by testing our hypotheses in a different country context we had access to—Germany—on a different set of salient world events—an attack against a refugee—using a larger adult sample. Although the case description was not based on any true events, the scenario was very topical, as issues concerning immigration and asylum have been a top concern in Germany for years (Kotzur et al., 2022; Statista Research Department, 2024), with the far‐right making significant gains in the most recent elections of the European Parliament based on anti‐immigration and anti‐refugee campaigning (Marsh & Escritt, 2024).

### Method and materials

#### Data collection and participants

The study was conducted at a time of international news attention to migration‐related issues, including occurrences of intergroup violence between refugees and receiving‐society members. Thus, violent conflicts between refugees and receiving‐society members were a salient topic at the time of data collection, particularly among German citizens. Overall, 360 German participants were recruited through Prolific in exchange for a small monetary reward: *N* = 188 for Study 3a, *N* = 172 for Study 3b. The experiments received ethical clearance from the Research Office of King's College London, Nr. MRA‐23/24‐42701. Sample size was determined by sensitivity analysis, which indicated that *n* = 80 per cell (i.e. *N* = 160 in total per study) would allow us to find an effect size of .31 or greater with 80% power. We slightly over‐recruited for both studies in case excluding participants was necessary.

One of our pre‐registered inclusion criteria was that participants needed to pass two attention checks. Of the 188 participants in Study 3a, we excluded *n* = 2 participants who failed a simple attention check, for which we asked them to click one specific option on a 5‐point Likert scale. As an additional attention check, we used a multiple‐choice question to check basic engagement with the manipulation text. Our requirement was that participants should be able to correctly select two from a total of six factual statements, for example, who the victim was, and whether the victim was dead or alive. In addition, participants should not select any wrong statements.^1^ These exclusions resulted in a total sample of *N* = 179 for Study 3a, and *N* = 157 for Study 3b. The analysed sample was 49% female, and 33 years old on average (SD = 11.79) in Study 3a, and 52% female and 31 years old on average (SD = 9.47) in Study 3b.

#### Procedure and measures

Following the procedure of previous studies, participants read a short description about the death of the Syrian refugee Mahmoud Aboud and Gerold Müller's role in his death (both had masculine names). In this scenario, Aboud was involuntarily involved in a physical fight with a German person in a park and was wrongly identified as the troublemaker by onlooker Müller. Müller was said to have intervened in the fight and punched Aboud, who died as a result of the impact of his fall. The only difference between Studies 3a and 3b was that Müller was portrayed as a private citizen in Study 3a, and as a police officer in Study 3b (for further details on the scenario, see Web Appendix C: Survey Study 3 for the English translation, or Web Appendix D for the German version: Appendix S1). As in previous studies, participants in both studies were then asked to place a checkmark next to the crimes for which they believed Müller should be charged. These crimes were: No offence, Negligent assault, Intentional assault, Manslaughter and Murder. Each crime was accompanied by a detailed description of what constituted the crime. Participants also rated how much time in prison Müller should spend if convicted for each crime on the same 5‐point Likert scale, labelled 1 (*1 month–2 years*), 2 (*3–5 years*), 3 (*5–7 years*), 4 (*8–10 years*) and 5 (*10 or more years*).

After this initial assessment, participants were randomly assigned to one of two conditions. In the Remorse condition, Müller offered a remorseful apology for contributing to Aboud's death, and as in Studies 1 and 2, signalled respect and concern for the victim by taking responsibility, expressing regret for their actions and the harm they have caused, and wishing for social cohesion. Participants in this condition read (translation of German text):

I am sorry about your son. I didn't realise how much aggression refugees have to experience in their daily lives. I feel so terrible. I thought I was helping and I never intended to cause serious harm. I am deeply sorry and wish I could make up for my mistake. I am learning a lot and just hope that somehow we can learn to accept each other through this terrible loss.

In the No Remorse condition, Müller did not express remorse for contributing to Aboud's death, deflecting responsibility and offering alternative justifications for their behaviour:

I am sorry about your son. I didn't realise he wasn't the attacker this time. I mean, you hear about people being attacked by people like him all the time, and I was just trying to help protect my community. I don't know what he was doing in the park, and I didn't even hit him very hard, it was just really bad luck that he fell so hard, but I don't think I'm much to blame.

Participants then rated how severely they would punish Müller on a scale from 1 (*very lenient*) to 7 (*very strict*) if they were a judge. In contrast to the previous two studies, participants were asked to be a judge instead of a juror due to differences in the judicial system (since juries do not exist in Germany). At the end of the study, participants completed the German 16‐item SDO7 scale (Saldarriaga et al., 2017, alpha = .94), a 4‐item attitude towards refugees scale (adapted from (Heitmeyer et al., 2013), alpha = .90) and basic demographic information. The attitude towards refugees scale included items like ‘Most refugees have no real fear of persecution in their home countries’.

### Results

#### Before the experimental manipulation

Table 7 lists, for each crime, the percentage of participants who believed Müller should be charged and the degree of punishment he should experience if convicted, for both Study 3a and Study 3b. Table 7 also lists the correlations for recommending charging Müller with each crime and the prescribed length of prison time with social dominance orientation for both studies.

**TABLE 7:** Type of crime, percentage of participants who checked each crime and the correlations of these choices with social dominance orientation before the experimental manipulation, Study 3a (individual actor) and Study 3b (state actor).

Type of Crime	Percentage checked	Punishment	Correlations with SDO	
			Checked	Punishment
No offence (may be wrong, but not illegal)				
Individual actor	2%	–	.15*	–
State actor	2%	–	.13^ns^	–
Negligent assault (resulting in death)				
Individual actor	23%	2.52 (1.18)	.10 ^ns^	−.10 ^ns^
State actor	29%	2.56 (1.28)	.24**	−.21**
Intentional assault (resulting in death)				
Individual actor	69%	3.38 (1.05)	−.02 ^ns^	−.11 ^ns^
State actor	62%	3.36 (1.09)	−.20**	−.21**
Manslaughter (intentional killing, without racism)				
Individual actor	17%	4.00 (0.95)	‐.08^ns^	−.12 ^ns^
State actor	17%	4.02 (0.95)	‐.13^ns^	−.21**
Murder (intentional homicide, with racism)				
Individual actor	21%	4.76 (0.69)	−.17*	–.13^ns^
State actor	20%	4.82 (0.52)	‐.07^ns^	‐.04^ns^

*Note*: *p < .05, **p < .01 and ***p < .001. Punishment indicates how lenient (1) or strict (7) a judge should be on the individual actor (Study 3a) and state actor (Study 3b), with means presented with standard deviations in parentheses. *N* = 87.

Overall, SDO did not consistently significantly correlate with indicating which crimes Müller should be charged with nor with the punishment Müller should receive if convicted in both studies. Thus, before our experimental manipulation, SDO did not play a consistent role in judging whether crimes were committed against Mahmoud Aboud, a Syrian refugee.

#### After the experimental manipulation

For both studies, we again conducted a multiple regression analysis with condition, attitudes towards refugees, SDO and the interaction between condition and SDO predicting the severity of punishment that Müller should experience if convicted. Table 8 displays the descriptive statistics, including correlations, among the variables used in the regression analyses in Study 3a and Study 3b.

**TABLE 8:** Means, standard deviations and correlations among variables included in the regression analysis, Study 3a (individual actor, *N* = 179) and Study 3b (state actor, *N* = 157).

Variables	*M*	SD	2.	3.	4.
1. Condition					
Individual actor	0.51	0.50	−.14	−.06	−.11
State actor	0.52	0.50	−.07	.09	−.26**
2. SDO					
Individual actor	2.48	1.03	–	.67***	−.29***
State actor	2.57	1.02		.67***	−.27***
3. Attitudes					
Individual Actor	3.77	1.60		–	−.32***
State actor	3.74	1.54			−.36***
4. Punishment					
Individual actor	5.13	1.39			–
State actor	5.27	1.25			

*Note*: *p < .05, **p < .01 and ***p < .001. Condition was coded such that 1 = Remorse, 0 = No Remorse response from Müller. Attitudes = Negative attitudes against refugees. Punishment indicates how lenient (1) or strict (7) Müller should be punished. Individual actor results stem from Study 3a; state actor results stem from Study 3b.

Abbreviation: SDO, social dominance orientation.

Consistent with Study 1 and Study 2, as well as previous research and our pre‐registration, SDO correlated positively with negative attitudes towards refugees, and negative attitudes correlated negatively with severity of punishment recommended for Müller in both samples. Hence, as in Studies 1 and 2, participants higher on group prejudice deprioritized punishing someone who harmed a member of a subordinated group.

Table 9 displays the results of the regression analysis. Overall, the regression models were statistically significant: for Study 3a, *F* (4, 174) = 7.89, *p* < .001, explaining 13.4% of the variance in severity of punishment, and for Study 3b, *F* (4, 152) = 10.70, *p* < .001, explaining 19.9%. We found statistically significant main effects of condition and attitudes towards refugees on severity of punishment across studies. People in the remorse condition (Study 3a: *b* = −1.37, SE = 0.52, *p* = .009; Study 3b: *b* = −1.67, SE = 0.49, *p* = .001) and people with more negative attitudes towards refugees (Study 3a: *b* = −0.18, SE = 0.08, *p* = .029; Study 3b: *b* = −0.21, SE = 0.08, *p* = .012) recommended more lenient punishments. We also found a main effect of SDO such that participants who scored higher in SDO endorsed more lenient punishments (Study 3a: *b* = −0.40, SE = 0.16, *p* = .014; Study 3b: *b* = −0.36, SE = 0.15, *p* = .023).

**TABLE 9:** Multivariate regression analyses of punishment, Study 3a (individual actor) and Study 3b (state actor).

Predictors	Individual actor		State actor	
	*b*	SE	*b*	SE
0. Intercept	7.02***	0.37	7.28***	0.36
1. Condition	−1.37**	0.52	−1.67***	0.49
2. SDO	−0.40**	0.16	−0.36*	0.15
3. Condition × SDO	0.39*	0.19	0.41*	0.18
4. Attitudes	−0.18*	0.08	−0.21*	0.08
Observations	179		157	
*R* ^2^ [*R* ^2^_adj_]	15.4% [13.4%]		22.0% [19.9%]	

*Note*: *p < .05, **p < .01 and ***p < .001. Condition was coded such that 1 = Remorse, 0 = No Remorse response from Müller. Attitudes = negative attitudes against refugees. Outcome variable is Punishment, indicating how lenient (1) or strict (7) Müller should be punished. Individual actor results stem from Study 3a; state actor results stem from Study 3b.

Abbreviation: SDO, social dominance orientation.

**FIGURE 2:**
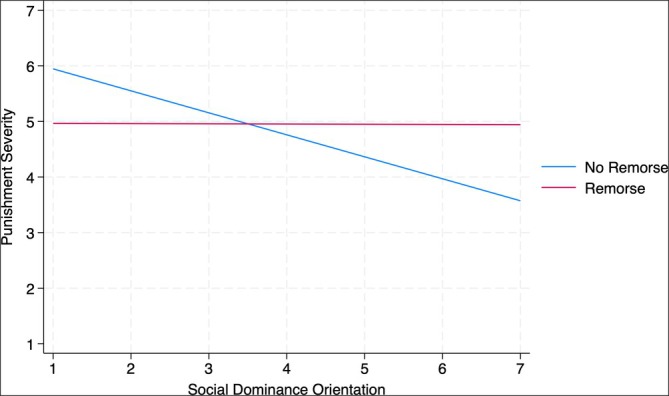
Interaction between social dominance orientation and the experimental manipulation predicting severity of punishment for individual actor (Study 3a, Simple Slopes).

**FIGURE 3:**
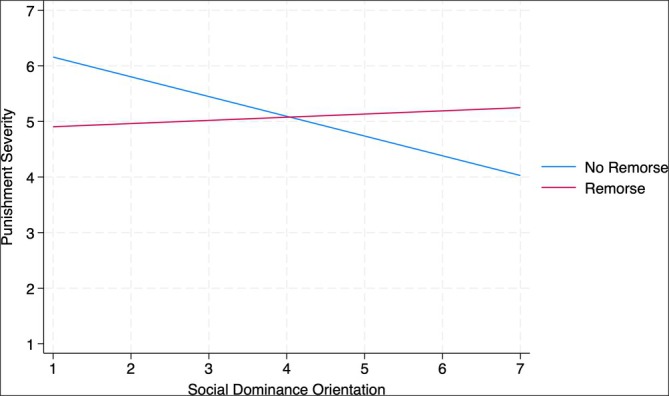
Interaction between social dominance orientation and the experimental manipulation predicting severity of punishment for state actor (Study 3b, Simple Slopes).

In addition, as predicted, we found the hypothesized interaction between SDO and our experimental manipulation in both Studies 3a and 3b (see Figures 2 and 3; Study 3a: *b* = 0.39, SE = 0.19, *p* = .045; Study 3b: *b* = 0.41, SE = 0.18, *p* = .021).

When Müller truly apologized for his actions displaying remorse, the SDO effect was less pronounced, suggesting that people who were lower in SDO endorsed less severe punishment, and people who were higher in SDO endorsed stricter punishment for Müller than their counterparts in the no remorse condition. Specifically, as can be seen in the simple slopes, in the no remorse condition, there was a significant negative relationship between SDO and punishment across both studies (Study 3a: *b* = −0.40, SE = 0.16, *p* = .014; Study 3b: *b* = −0.36, SE = 0.15, *p* = .023). However, in the remorse condition, the effect of SDO on punishment was not significant (Study 3a: *b* = −0.004, SE = 0.16, *p* = .982; Study 3b: *b* = 0.06, SE = 0.14, *p* = .692).

### Discussion

Study 3a and Study 3b were conducted to reconcile the conflicting results between Study 1 and 2, and to test our hypotheses with a different group being victimized and, for the first time, another country context than the previous studies—an alleged crime against a Syrian refugee by a German in Germany. Our manipulation was successful regardless of whether the perpetrator was a private citizen (Study 3a) or a police officer (Study 3b), and thus acted on behalf of the state. In addition, like in Study 2, we found a significant main effect of SDO, suggesting that higher SDO levels were associated with less punitive attitudes. Importantly, we were able to find support for our pre‐registered interaction hypothesis. Like in Study 1, we found that high SDO levels enhanced punitive attitudes after learning about the perpetrators' remorse in an apology, whereas low levels of SDO reduced them.

These results do not support the suggestions that the level of power and authority afforded to the perpetrator—acting on behalf of the state versus private individual—might explain the differences in the result patterns across the previous two studies. Indeed, the effect sizes of the interaction terms were nearly identical in Studies 3a and 3b. Instead, it might have been the improved study design—including attention checks—that made the difference by ensuring that participants' engagement with the study materials remained high, resulting in a similar results pattern to those in Study 1.

## MINI META‐ANALYSIS ACROSS ALL STUDIES

Given the heterogeneity of our findings, we conducted a mini‐meta‐analysis across all interactions between condition and SDO from the final models. We followed Goh et al. (2016) to obtain the overall mean interaction effect and tested for significance of the effect using both a fixed‐effect and random‐effect approach. Across all studies, the overall weighted mean interaction effect was .47. The effect was significant using both a fixed‐effect approach, combined *z* = 10.60, *p* < .001, and a random‐effect approach, *t* (3) = 6.55, *p* = .004. A chi‐square test for heterogeneity was non‐significant, χ^2^(33) = 13.31, *p* = .999, suggesting no substantial variability in effect sizes across studies.

## GENERAL DISCUSSION

Ravi never apologized to Clementi's family during the court hearing. Indeed, the judge in the case addressed him directly, stating, ‘I heard this jury say, “guilty” 288 times […], I haven't heard you apologize once’ (Bratu, 2012). When Ravi finally released a statement after being scolded by the judge, Clementi's family noted that ‘His press release did not mention Tyler or our family, and it included no words of sincere remorse, compassion or responsibility for the pain he caused’ (Mulvihill & Henry, 2012). This highlights the complex tension around apologies and remorse's role in resolving harm, especially in situations where power imbalances shape perceptions of accountability and justice. Opinions diverge on whether the powerful are justified in exercising their power against the less powerful, whether resulting harm is inherently criminal or unjust and whether past abuses warrant punitive measures or sincere apologies. At the global stage, we observe a stark duality: While some powerful groups have taken steps towards accountability by issuing formal apologies for historical wrongs, others use their power to perpetuate harm and oppression against subordinate groups. For example, institutions have formally apologized for systemic violence, including child abuse, sexual abuse and other atrocities once perpetrated with impunity against less powerful populations (e.g. Lind, 2008). Yet, in contrast, we are witnessing a resurgence of forceful territorial acquisitions, the curtailment of Indigenous rights and the suppression of ethnic and other minority groups. In both intergroup settings and in courts of law, questions arise of whether suffering by subordinated persons implies a crime, and whether apologies indicating remorse for causative actions reduce punishments. Understanding the psychological processes at play in such decisions can inform us about short‐term recreation of intergroup relations, potential biases and presumptions that affect criminal justice. We summarize how our results extend prior research before outlining their limitations and directions for future research.

We predicted that a remorseful intergroup apology (vs. no remorseful apology) will moderate the effect of SDO on participants' perceived justified severity of punishment after a possible hate crime. We tested our predictions across three different settings and victim social categories—presumptive psychological and social harm to a gay man (Study 1), death from gun violence for a Black man (Study 2) and death from physical altercation for a refugee (Studies 3a and 3b). Our internal mini‐meta‐analysis revealed that, overall, the hypothesized interaction effect was significant, lending confidence and robustness to our findings. As such, our study offers important insights into the complexity of moral judgements, underscoring the important and nuanced contribution of ideology colouring punitive attitudes.

Across three of four studies (Studies 1, 3a and 3b), people who desired group dominance advocated harsher punishment of the perpetrator who expressed remorse than did people who desired group‐based equality, even after controlling for negative attitudes towards the subordinate group. This suggests that people who are high on SDO become more punitive towards people who thwart the goal of group dominance. What is more, people low on SDO become more forgiving and less punitive once the dominant group member expressed remorse (Studies 1, 3a and 3b). This was regardless of whether the study was carried out with a student sample in the United States (Study 1) or a heterogeneous adult sample in Germany (Studies 3a and 3b). This indicates the effect is not highly limited. In addition, the use of experimental designs across all of our studies manipulating the display of remorse lends high internal validity to our findings. The fact that Study 2 did not produce a statistically significant interaction effect may be due to design‐related limitations. Specifically, Study 2 lacked attention checks and may have suffered from reduced participant engagement, introducing additional variance and decreasing statistical power. While the direction and size of the estimated interaction were broadly consistent with those found in the other studies, the estimate was not significant. The internal mini meta‐analysis revealed no significant heterogeneity in effect sizes across studies, suggesting that the pattern of results was robust despite the non‐significant finding in Study 2.

Extending prior research, we found a strong main effect of SDO on punishment in Study 2, 3a and 3b, such that those *lower* in SDO supported stricter punishments for the perpetrator than those higher in SDO. This effect contrasts with previous research in which individuals endorsing hierarchies are typically *more* punitive (Green et al., 2009; Pratto et al., 1998). For instance, previous survey research revealed that individuals higher in SDO are more supportive of harsher sanctions in the United States (Sidanius et al., 2006). Here, when low SDO people recommended harsher punishments, they did so when a member of a dominant group had caused death to a member of a subordinate group. Nonetheless, the mixture of prior and present findings supports our contention that SDO alone is not enough to determine a person's recommended punishment severity.

We suggest that the present main effect that low SDO people endorsed more punishment than high SDO people was previously not uncovered because prior research asked about the most typical types of punishment. In contrast, our scenarios described violence by a dominant group member against a subordinate group member—an act that can amount to a hate crime—which might have sufficed as signalling the hierarchy‐enhancing meaning of these acts for relations between dominants and subordinates. Importantly, though, our results did show that, as we suggested, a gesture of remorse by the alleged perpetrator following the events was taken by people low on SDO to signal that social relations can be repaired with lenience (Studies 1, 3a and b); they, in contrast, show that to people higher on SDO, to be remorseful following an act of harm signalled, as expected, that the perpetrator is not being a real hierarchy‐enhancer, and therefore deserves harsher punishment (Studies 1, 3a and b).

A practical implication here concerns members of dominant groups who are charged with crimes against members of subordinate groups—should they appear remorseful? Our results suggest that it depends on whom they want to please. For policymakers, especially in the justice system, recognizing that not everyone responds to remorse in the same way could lead to more nuanced approaches in sentencing and rehabilitation programmes. Thus, our study highlights the need for diverse strategies that consider individual differences in this area.

There are many ways future studies can build on ours. Firstly, although we have shown that our results hold for both the United States and Germany, our investigations have been limited to two specific country contexts. What is more, although we have varied the acts of harm and target groups under investigation, we have limited ourselves to testing three different victim groups and acts. Although we cannot think of any theoretical reason why these results should not reproduce for other members of subordinate groups, or other (violent) acts of harm, future research could expand on our research by delivering a broader empirical base for our theorizing. Especially expanding towards other cultures that vary in individuals' perceptions of authorities and beliefs about the punishment system, seems worthwhile to us.

Moreover, across all scenarios we have used, the alleged crimes were of a very serious nature, which ultimately resulted in the death of the victim. There is reason to believe that the seriousness of the alleged crime might moderate the effects reported here, which future research could test empirically. Additionally, we have primarily focused on dominant group members' views in the present study. Social dominance theory suggests that subordinate members sometimes collaborate in their oppression (e.g. Sidanius & Pratto, 1999), which is supported by empirical studies that have shown healthy variance in the extent to which subordinate members prefer hierarchies or choose hierarchy‐enhancing careers (e.g. Sidanius et al., 2006). On the other hand, people in subordinated groups are, on average, lower on SDO than people in dominant groups (Lee et al., 2011) and they may have hierarchy‐attenuating legitimizations from which to make judgements about justice. Further testing will show whether results replicate for subordinate group members.

Our operationalization of a truthfully remorseful apology in our experimental manipulations across all studies included multiple components, such as that the alleged perpetrator took responsibility, expressed regret for their actions and harm caused, and wished for social cohesion, whereas the perpetrators in our control conditions deflected responsibility and justified their behaviour. Since all of the manipulations followed this basic structure, yet we allowed for slight variation in which way these components manifest depending on the transgression at hand, we cannot disentangle which of these components of a truthfully (non‐)remorseful apology are essential and which of them we could do without, which we would encourage future research to discern.

What is more, Study 2 results did not support our core interaction hypothesis. As mentioned before, we believe that this is likely due to methodological shortcomings of the study, which resulted in participants' reduced engagement with the study materials. Nonetheless, further studies advancing our understanding of further boundary conditions and extending our understanding by investigating further explanations for why people higher in SDO become more punitive towards dominant group members after offering remorseful apologies would be highly desirable.

Though all our studies were conducted during salient discussions that were highly related to the topics of our investigation, the sample sizes of Studies 1 and 2 were primarily driven by what our research budgets allowed at the time. Future studies could build on ours by replicating and extending our results with larger samples to receive more accurate effect size estimates for the populations studied.

## CONCLUSIONS

Across four studies, we found evidence for our postulate that the extent to which a person endorses hierarchies affects their punitive attitudes after signals of remorse. With the exception of Study 2, we consistently found that people higher in SDO feel that an alleged harmer of a person in a subordinated group should be punished less severely after offering a remorseful apology, whereas individuals lower in SDO feel that the punishment should be stricter. Our internal mini meta‐analysis offers further confidence in the robustness of our findings. Our results contribute to the literature on apologies, social dominance theory, and legal and social psychology in general, and thus to the understanding of the psychology behind the conditionality of people's senses of what harm should be punished.

## AUTHOR CONTRIBUTIONS

**Andrés Gvirtz:** Conceptualization; methodology; data curation; investigation; formal analysis; project administration; resources; visualization; writing – original draft; writing – review and editing; validation. **Patrick F. Kotzur:** Conceptualization; formal analysis; methodology; writing – original draft; writing – review and editing; investigation. **Andrew L. Stewart:** Conceptualization; methodology; data curation; investigation; formal analysis; writing – review and editing; writing – original draft; resources; funding acquisition. **Felicia Pratto:** Conceptualization; data curation; formal analysis; investigation; methodology; funding acquisition; resources; writing – original draft; writing – review and editing.

## CONFLICT OF INTEREST STATEMENT

The authors declare no conflicts of interest.

## Supporting information


Appendix S1.

## Data Availability

Data and analysis codes for all studies, as well as the pre‐registration for Studies 3a and 3b, can be found here: https://osf.io/kjs92/?view_only=2a7c4aedf2b1475ab62eb4c2a21758f5.

## Footnotes

^1^
We originally also had included additional response options, asking participants who the ‘indicted’ individual in the scenario was (Müller or Aboud), and have pre‐registered that participants should select ‘Müller’ to be included in the sample. However, the German translation of the scenario mentioned only that the state is *considering* whether to indict Mr. Müller, not that they *had* indicted him. As a result, a large proportion of the German participants (*n* = 32) correctly identified the victim and what had happened, but left the box about who had been indicted empty, which is formally—and in hindsight—the correct answer. As going by what would formally be correct for the third scenario based on attention checks would result in deletion of 85% of participants, and excluding those who had been formally correct would not make much sense and lead to a deletion of 9% of participants, both options were accepted.
