# Supplementary material for: Remorse for discrimination: The role of group dominance in judging hate crimes against subordinate group members
Source: Br J Soc Psychol. 2025 Sep 2;64(4):e70008. doi: 10.1111/bjso.70008 (PMC12405740; doi:10.1111/bjso.70008)
Supplement: Supplementary file 1 — Appendix S1. [file BJSO-64-0-s001.docx]

**Web Appendix**

**Remorse for Discrimination:**

**The Role of Group Dominance in Judging Crimes against Subordinate Groups**

| **Table of Contents** |
| --- |
| Web Appendix A: Survey Study 1 2-6 |
| Web Appendix B: Survey Study 2 7-14 |
| Web Appendix C: Survey Study 3: English Translation 15-23 |
| Web Appendix D: Survey Study 3: German Original 24-33 |

**Web Appendix A: Survey Study 1**

Tyler Clementi was a freshman student at Rutgers University when his roommate (Dharun Ravi) secretly videotaped Clementi having sex with a man. After his roommate posted the video on the internet without Clementi’s knowledge or consent, Clementi decided to commit suicide by jumping off of the George Washington Bridge on September 22, 2010. New Jersey prosecutors are now seeking to charge Ravi with some crime.

Pretend that you are a jury member in a trial against Dharun Ravi for secretly videotaping his roommate having sex with a man and posting it without his consent on the internet and possibly contributing to Clementi’s suicide. For which crime or crimes should Ravi be held responsible? Place a check next to all that you think Ravi should be tried for. The descriptions of the crimes are listed next to each possible charge.

| Check here | Potential Charge | Description of the Charge |
| --- | --- | --- |
|  | No Crime | What Ravi did was wrong but not illegal |
|  | Invasion of Privacy | Unlawfully intruding into someone’s private affairs, disclosing their private information, or publicizing them in a false light |
|  | Cyber bullying | Involves the use of information and communication technologies to support deliberate, repeated, and hostile behavior by an individual or group, that is intended to harm others |
|  | Hate crime | Act of violence against someone because of the group they belong to |
|  | Manslaughter | Killing someone with no intent at all of actually killing them |
|  | Second Degree Murder | Killing someone without planning to do so in advance but intending to do so in the moment |
|  | First Degree Murder | Killing someone with planning beforehand and carrying the plan out |

If Ravi is convicted with invasion of privacy, how much time in prison should he serve?

1 month to 2 years 3 to 5 years 5 to 7 years 8 to 10 years 10 or more years

If Ravi is convicted with cyber bullying, how much time in prison should he serve?

1 month to 2 years 3 to 5 years 5 to 7 years 8 to 10 years 10 or more years

If Ravi is convicted with a hate crime, how much time in prison should he serve?

1 month to 2 years 3 to 5 years 5 to 7 years 8 to 10 years 10 or more years

If Ravi is convicted with manslaughter, how much time in prison should he serve?

1 month to 2 years 3 to 5 years 5 to 7 years 8 to 10 years 10 or more years

If Ravi is convicted with second degree murder, how much time in prison should he serve?

1 month to 2 years 3 to 5 years 5 to 7 years 8 to 10 years 10 or more years

If Ravi is convicted with first degree murder, how much time in prison should he serve?

1 month to 2 years 3 to 5 years 5 to 7 years 8 to 10 years 10 or more year

Ravi made a statement to Clementi’s family:

“I’m sorry about your son. I didn’t know he was so fragile. It just seemed to me that if he didn’t mind getting it on with a man, he wouldn’t mind everyone knowing about it. I don’t see why anyone thinks this is my fault. Either you’re a man, or you’re not. I know what I am.”

Assuming Ravi is convicted of a crime, how much would you advise the judge to punish Ravi?

Circle how strict or lenient the judge should be on the scale below:

Very lenient 1 2 3 4 5 6 7 Very strict

Check the box above the most appropriate sentence below:

|  |  |  |  |  |  |  |
| --- | --- | --- | --- | --- | --- | --- |
| Suspended sentence (no punishment) | Small fine | Medium-sized fine | Large fine | Short prison sentence | Medium prison sentence | Long prison sentence |

Ravi made a statement to Clementi’s family:

“I’m sorry about your son. I had no idea my actions would want to make him take his own life. I feel so terrible. I didn’t realize before that being gay could be so painful. I am learning a lot and I just hope that somehow, through this awful loss, we can learn to accept one another.”

Assuming Ravi is convicted of a crime, how much would you advise the judge to punish Ravi?

Circle how strict or lenient the judge should be on the scale below:

Very lenient 1 2 3 4 5 6 7 Very strict

Check the box above the most appropriate sentence below:

|  |  |  |  |  |  |  |
| --- | --- | --- | --- | --- | --- | --- |
| Suspended sentence (no punishment) | Small fine | Medium-sized fine | Large fine | Short prison sentence | Medium prison sentence | Long prison sentence |

How much do you agree or disagree with the following statements? Please indicate your agreement or disagreement using the following scale from 1 (strongly disagree) to 7 (strongly agree).

| Strongly Disagree | 1 | 2 | 3 | 4 | 5 | 6 | 7 | Strongly Agree |
| --- | --- | --- | --- | --- | --- | --- | --- | --- |

1. Some groups of people must be kept in their place.
2. It’s probably a good thing that certain groups are at the top and other groups are at the bottom.

3. An ideal society requires some groups to be on top and others to be on the bottom.

4. Some groups of people are simply inferior to other groups.

5. Groups at the bottom are just as deserving as groups at the top.

6. No one group should dominate in society.

7. Groups at the bottom should not have to stay in their place.

8. Group dominance is a poor principle.

9. We should not push for group equality.

10. We shouldn’t try to guarantee that every group has the same quality of life.

11. It is unjust to try to make groups equal.

12. Group equality should not be our primary goal.

13. We should work to give all groups an equal chance to succeed.

14. We should do what we can to equalize conditions for different groups.

15. No matter how much effort it takes, we ought to strive to ensure that all groups have the same chance in life.

16. Group equality should be our ideal.

1. Sex between two men is just plain wrong.
2. I think male homosexuals are disgusting.
3. Male homosexuality is a natural expression of sexuality in men.
4. Sex between two women is just plain wrong.
5. I think lesbians are disgusting.
6. Female homosexuality is a natural expression of sexuality in women.

**Web Appendix B: Survey Study 2**

An African-American man, Anthony Taylor, was walking back to his apartment, when a police officer stopped him. The White officer, Luke Clark, reported afterwards that Taylor’s apparel fit an open warrant from another incident. When officer Clark asked Taylor to show his ID, Taylor reacted with irritation. When Officer Clark left the vehicle, Taylor started to move toward him. Officer Clark urged Taylor to stop immediately. When Taylor went to take something out of his pocket, the officer shot unarmed Taylor in the chest. Taylor was pronounced dead at the scene. State prosecutors are now seeking to charge Officer Taylor with some crime.

Pretend that you are a jury member in a trial against the Officer Luke Clark for shooting Anthony Taylor. For which crime or crimes should officer Clark be held responsible? Place a check next to all that you think Officer Clark should be tried for. The descriptions of the crimes are listed next to each possible charge.

| Check here | Potential Charge | Description of the Charge |
| --- | --- | --- |
|  | No Crime | What officer Clark might be wrong but not illegal |
|  | Hate crime | Act of violence against someone because of the group they belong to |
|  | Involuntary Manslaughter | Killing someone with no intent at all of actually killing them, but through being reckless |
|  | Voluntary Manslaughter | Killing someone with no intent at all of actually killing them, but due to circumstances that explain emotionally disturbance |
|  | Second Degree Murder | Killing someone without planning to do so in advance but intending to do so in the moment |
|  | First Degree Murder | Killing someone with planning beforehand and carrying the plan out |

If officer Clark is convicted with a hate crime, how much time in prison should he serve?

1 month to 2 years 3 to 5 years 5 to 7 years 8 to 10 years 10 or more years

If officer Clark is convicted with manslaughter, how much time in prison should he serve?

1 month to 2 years 3 to 5 years 5 to 7 years 8 to 10 years 10 or more years

If officer Clark is convicted with second degree murder, how much time in prison should he serve?

1 month to 2 years 3 to 5 years 5 to 7 years 8 to 10 years 10 or more years

If officer Clark is convicted with first degree murder, how much time in prison should he serve?

1 month to 2 years 3 to 5 years 5 to 7 years 8 to 10 years 10 or more yea

Officer Clark made a statement to Taylor’s Family:

“I’m sorry about your son. If I would be back in the situation, I would warned him louder, hoping that he didn’t move so that I didn’t have to assume he was pulling out a weapon. I can’t really see why anyone thinks this is my fault. As an officer in the line of duty your life is threatened all the time, you have to make decisions.

Assuming Officer Clark is convicted of a crime, how much would you advise the judge to punish him?

Very lenient 1 2 3 4 5 6 7 Very strict

Check the box above the most appropriate sentence below:

|  |  |  |  |  |  |  |
| --- | --- | --- | --- | --- | --- | --- |
| Suspended sentence (no punishment) | Small fine | Medium-sized fine | Large fine | Short prison sentence | Medium prison sentence | Long prison sentence |

“I’m sorry about your son. I felt so terrible when I realized that he didn’t have a gun with him. I never intended to take his life. It was a split-second decision, I thought my life is endangered. I feel deeply sorry about what happened and wished I could redo my mistake. I offer my sincere condolences.

Assuming Officer Clark is convicted of a crime, how much would you advise the judge to punish him?

Circle how strict or lenient the judge should be on the scale below:

Very lenient 1 2 3 4 5 6 7 Very strict

Check the box above the most appropriate sentence below:

|  |  |  |  |  |  |  |
| --- | --- | --- | --- | --- | --- | --- |
| Suspended sentence (no punishment) | Small fine | Medium-sized fine | Large fine | Short prison sentence | Medium prison sentence | Long prison sentence |

Instructions. How much do you agree or disagree with the following statements? Please indicate your agreement or disagreement using the following scale from 1 (strongly disagree) to 7 (strongly agree).

| Strongly Disagree | 1 | 2 | 3 | 4 | 5 | 6 | 7 | Strongly Agree |
| --- | --- | --- | --- | --- | --- | --- | --- | --- |

| 1. | If a black were put in charge of me, I would not mind taking advice and direction from him or her. |
| --- | --- |
| 2. | If I had a chance to introduce black visitors to my friends and neighbors, I would be pleased to do so. |
| 3. | I would rather not have blacks live in the same apartment building I live in. |
| 4. | I would probably feel somewhat self conscious dancing with a black in a public place. |
| 5. | I would not mind it at all if a black family with about the same income and education as me moved in next door. |
| 6. | I think that black people look more similar to each other than white people do. |
| 7. | Interracial marriage should be discouraged to avoid the “who-am-I?” confusion which children feel. |
| 8. | I get very upset when I hear a white make a prejudicial remark about blacks. |
| 9. | I favor open housing laws that allow more racial integration of neighborhoods. |
| 10. | It would not bother me if my new roommate was black. |
| 11. | It is likely that blacks will bring violence to neighborhoods when they move in. |
| 12. | I enjoy a funny racial joke, even if some people might find it offensive |
| 13. | The federal government should take decisive steps to override the injustices blacks suffer at the hands of local authorities. |
| 14. | Black and white people are inherently equal. |
| 15. | Black people are demanding too much too fast in their push for equal rights. |
| 16. | Whites should support blacks in their struggle against discrimination and segregation. |
| 17. | Generally, blacks are not as smart as whites. |
| 18. | I worry that in the next few years I may be denied my application for a job or a promotion because of preferential treatment given to minority group members. |
| 19. | Racial integration (of schools, businesses, residences, etc.) has benefitted both whites and blacks. |
| 20. | Some Blacks are so touchy about race that it is difficult to get along with them. |

1. Some groups of people must be kept in their place.

2. It’s probably a good thing that certain groups are at the top and other groups are at the bottom.

3. An ideal society requires some groups to be on top and others to be on the bottom.

4. Some groups of people are simply inferior to other groups.

5. Groups at the bottom are just as deserving as groups at the top.

6. No one group should dominate in society.

7. Groups at the bottom should not have to stay in their place.

8. Group dominance is a poor principle.

9. We should not push for group equality.

10. We shouldn’t try to guarantee that every group has the same quality of life.

11. It is unjust to try to make groups equal.

12. Group equality should not be our primary goal.

13. We should work to give all groups an equal chance to succeed.

14. We should do what we can to equalize conditions for different groups.

15. No matter how much effort it takes, we ought to strive to ensure that all groups have the same chance in life.

16. Group equality should be our ideal.

This study is anonymous, but we would like to be able to summarize the characteristics of people who participated. Please describe yourself by checking or completing the appropriate blanks.

Your gender: Male _____ Female _____ Your age: _____ years

Year of study: ___ First Year ___ Sophomore ___ Junior ___ Senior ___ Not Applicable

Which ethnic group do you belong to?

_____ Black/African American _____ Asian/Asian American

_____ White/Euro-American _____ Latino/Hispanic American

_____ Middle Eastern/Arab American _____ Native American

Other (please specify) _______________________________________________________________

Do you consider the United States to be your native country? _____ yes _____ no (If not, specify:_____

Are you a United States citizen? ______ yes _____ no

What is your religion?

____ Catholic _____ Protestant _____ Jewish _____ Muslim

____ Buddhist _____ Hindu _____ Atheist _____ Other (please specify)_____________

What is your family's social class position?

_____ Poor

_____ Working Class

_____ Middle Class

_____ Upper Middle Class

_____ Upper Class

Check your mother (or guardian’s) highest education level: And your father (or guardian’s):

_____ No Formal Education _____ No Formal Education

_____ Elementary School _____Elementary School

_____ Some High School _____ Some High School

_____ Completed High School _____ Completed High School

_____ Some College _____ Some College

_____ BA or BS Degree _____ BA or BS Degree

_____ Some Graduate/Professional School _____ Some Graduate/Professional School

_____ Hold Graduate/Professional Degree _____ Hold Graduate/Professional Degree

How Liberal or Conservative are you on: (write a number from 1 to 7 using the scale below)

_____ foreign policy issues? ____ economic issues? ____ domestic issues?

Very conservative 1 2 3 4 5 6 7 Very liberal

How much do you follow the news?

Not at all 1 2 3 4 5 6 7 Very Much

From where do you get your news (Circle all that apply)?

Newspapers Cable TV: CNN Cable TV: Fox News Cable TV: MSNBC

Radio: NPR Network TV: CBS Network TV: NBC Network TV: ABC

Internet: Please list website you use most for news ______________________

**Web Appendix C: Survey Study 3 English Translation**

Condition 1A:

Please read the following article on the course of events of a possible criminal offense carefully (some follow-up questions refer to this article):

Syrian refugee Mahmud Aboud was walking through a park when he was bumped into by drunk Max Schmidt, who began arguing with Mahmud Aboud. Gerold Müller, who was walking through the park, saw the two arguing, assumed that Max Schmidt was the victim and knocked Mahmud Aboud unconscious, whereupon he fell to the sidewalk and died as a result of the impact. A surveillance camera in the park later confirmed that Max Schmidt attacked Mahmud Aboud without prior provocation and that Gerold Müller appeared two minutes after the altercation began and delivered the fatal blow to Mahmud Aboud. Investigations revealed that Gerold Müller is a member of chat groups in which colleagues exchange right-wing extremist messages. The public prosecutor's office is now considering charging Gerold Müller with a criminal offense.

Condition 1B:

Please read the following article on the course of events of a possible criminal offense carefully (some follow-up questions refer to this article):

Syrian refugee Mahmud Aboud was walking through a park when he was bumped into by drunk Max Schmidt, who began arguing with Mahmud Aboud. Police officer Gerold Müller, who was walking through the park, saw the two arguing, assumed that Max Schmidt was the victim and knocked Mahmud Aboud unconscious, whereupon he fell to the sidewalk and died as a result of the impact. A surveillance camera in the park later confirmed that Max Schmidt attacked Mahmud Aboud without prior provocation and that police officer Gerold Müller arrived two minutes after the confrontation began and dealt the fatal blow to Mahmud Aboud. Investigations revealed that police officer Gerold Müller is a member of chat groups in which colleagues exchange right-wing extremist messages. The public prosecutor's office is now considering charging Gerold Müller with a criminal offense.

What happened in the scenario just described? Please check all that apply.

- Syrian refugee Mahmud Aboud is the accused

- Syrian refugee Mahmud Aboud is the victim

- Gerold Müller is the defendant

- Gerold Müller is the victim

- The victim is deceased

- The victim is alive

Imagine being a judge in the trial against the defendant Gerold Müller for the death of the Syrian refugee Mahmud Aboud. On which charges should Gerold Müller be found guilty?

Please tick all the points on which you think Gerold Müller should be charged. The description of the offenses is next to the possible charges.

- No offense (what Gerold Müller did may be wrong, but not illegal)

- Negligent assault resulting in death (no intentional homicide or injury)

- Intentional assault resulting in death (no intentional homicide, but intentional injury - not necessarily due to racist motives)

- Manslaughter (intentional killing, without base motives, such as racism)

- Murder (intentional homicide, with base motives, such as racism)

If Gerold Müller is found guilty of negligent assault causing death, how long do you think the prison sentence should be?

- 1 month to 2 years

- 3 to 5 years

- 5 to 7 years

- 8 to 10 years

- 10 or more years

If Gerold Müller is found guilty of willful bodily harm resulting in death, how long do you think the prison sentence should be?

- 1 month to 2 years

- 3 to 5 years

- 5 to 7 years

- 8 to 10 years

- 10 or more years

If Gerold Müller is found guilty of manslaughter, how long do you think the prison sentence should be?

- 1 month to 2 years

- 3 to 5 years

- 5 to 7 years

- 8 to 10 years

- 10 or more years

If Gerold Müller is found guilty of murder, how long do you think the prison sentence should be?

- 1 month to 2 years

- 3 to 5 years

- 5 to 7 years

- 8 to 10 years

- 10 or more years

Condition 2A:

Gerold Müller has issued the following statement to the Abouds family:

I am sorry about your son. I didn't realise how much aggression refugees have to experience in their daily lives. I feel so terrible. I thought I was helping and I never intended to cause serious harm. I am deeply sorry and wish I could make up for my mistake. I am learning a lot and just hope that somehow we can learn to accept each other through this terrible loss.

Condition 2B:

Gerold Müller has issued the following statement to the Abouds family:

I am sorry about your son. I didn't realise he wasn't the attacker this time. I mean, you hear about people being attacked by people like him all the time, and I was just trying to help protect my community. I don't know what he was doing in the park, and I didn't even hit him very hard, it was just really bad luck that he fell so hard, but I don't think I'm much to blame.

Severity of the punishment:

Assuming you were a judge at the trial and found Gerold Müller guilty of a criminal offense, what verdict would you reach?

- Very lenient punishment (1)

- (2)

- (3)

- (4)

- (5)

- (6)

- Very severe punishment (7)

Check the sentence that seems most appropriate to you:

- Acquittal (no penalty)

- Small fine

- Medium fine

- Heavy fine

- Short prison sentence

- Medium prison sentence

- Long prison sentence

How much do you agree with the following statements?

Please indicate the extent to which you agree or disagree with the statements.

- Refugees living in Germany are a burden on the social system.

- There are too many refugees living in Germany.

- The state should be generous when examining asylum applications.

- Most refugees have no real fear of persecution in their home countries.

Strongly disagree - Strongly agree (7-point scale)

How much do you agree with the following statements? Please indicate the extent to which you agree or disagree with the statements.

- Some groups of people must be kept in their place.

- It’s probably a good thing that certain groups are at the top and other groups are at the

bottom.

- An ideal society requires some groups to be on top and others to be on the bottom.

- Some groups of people are simply inferior to other groups.

- Groups at the bottom are just as deserving as groups at the top.

- No one group should dominate in society.

- Groups at the bottom should not have to stay in their place.

- Group dominance is a poor principle.

- We should not push for group equality.

- We shouldn’t try to guarantee that every group has the same quality of life.

- It is unjust to try to make groups equal.

- Group equality should not be our primary goal.

- We should work to give all groups an equal chance to succeed.

- Please check "tend to agree" here. Thank you. (Attention check)

- We should do what we can to equalize conditions for different groups.

- No matter how much effort it takes, we ought to strive to ensure that all groups have the

same chance in life.

- Group equality should be our ideal.

Finally, please provide a few details about yourself.

Below you can see a ladder. At the top of the ladder are the people who are best off, who have the most money, the most education and the best jobs. At the bottom are the people who are worst off, who have the least money, the least education, the worst jobs or no work at all.

Please mark the rung that you think best represents where you are on the ladder.

- 1

- 2

- 3

- 4

- 5

- 6

- 7

Please specify your gender.

- Female

- Male

- Other, namely __________________________________________________

Age

Please enter your age (in years).

________________________________________________________________

Were you born in Germany?

- Yes

- No

Do you have German nationality?

- Yes

- No

Which religion do you feel you belong to?

- Catholicism/Christianity

- Judaism

- Islam

- Buddhism

- Hinduism

- None

Other (please specify) __________________________________________________

What is your highest general school-leaving qualification?

- Pupil at a general education school

- Left school without a school-leaving certificate

- Secondary school certificate, elementary school certificate or comparable qualification

- Secondary school certificate, intermediate school leaving certificate or comparable qualification

- Entrance qualification for universities of applied sciences

- Abitur, general or subject-specific higher education entrance qualification, grammar school

What is your highest vocational training qualification?

- Still in training, studying

- No vocational qualification and not in vocational training

- Apprenticeship (vocational training in a company)

- Vocational qualification from a vocational school

- Degree from a technical school, master craftsman school, administration and business academy, vocational or technical academy

- Bachelor's degree, diploma (FH)

- Master's degree, diploma (university), Magister, state examination, doctorate

Which party would you vote for if there were a general election next Sunday?

- SPD

- CDU/CSU

- Greens

- FDP

- AfD

- Left

- Others: __________________________________________________

Political orientation: In politics, people often talk about "left" and "right". Please indicate how left or right you would place yourself.

- Left (0)

- (1)

- (2)

- (3)

- (4)

- (5)

- (6)

- (7)

- (8)

- (9)

- Right (10)

**Web Appendix D: Survey Study 3 German**

Bedingung 1A:

Bitte lesen Sie folgenden Artikel zum Hergang einer möglichen Straftat aufmerksam durch (einige Fragen im Nachgang beziehen sich auf diesen Artikel):
Der syrische Flüchtling Mahmud Aboud ging durch einen Park, als er vom betrunkenen Max Schmidt angerempelt wurde und dieser mit Mahmud Aboud zu streiten begann. Gerold Müller, der durch den Park ging, sah die beiden streiten, nahm an, dass Max Schmidt das Opfer sei, und schlug Mahmud Aboud bewusstlos, woraufhin dieser auf den Bürgersteig fiel und an den Folgen des Aufpralls starb. Eine Überwachungskamera im Park bestätigte später, dass Max Schmidt Mahmud Aboud ohne vorangegangene Provokation angriff und dass Gerold Müller zwei Minuten nach Beginn der Auseinandersetzung erschien und Mahmud Aboud den tödlichen Schlag versetzte. Ermittlungen ergaben, dass Gerold Müller Mitglied von Chatgruppen ist, in denen Kollegen rechtsextremistische Nachrichten austauschen. Die Staatsanwaltschaft erwägt nun, Gerold Müller einer Straftat anzuklagen.

Bedingung 1B:

Bitte lesen Sie folgenden Artikel zum Hergang einer möglichen Straftat aufmerksam durch (einige Fragen im Nachgang beziehen sich auf diesen Artikel):
Der syrische Flüchtling Mahmud Aboud ging durch einen Park, als er vom betrunkenen Max Schmidt angerempelt wurde und dieser mit Mahmud Aboud zu streiten begann. Polizist Gerold Müller, der durch den Park ging, sah die beiden streiten, nahm an, dass Max Schmidt das Opfer sei, und schlug Mahmud Aboud bewusstlos, woraufhin dieser auf den Bürgersteig fiel und an den Folgen des Aufpralls starb. Eine Überwachungskamera im Park bestätigte später, dass Max Schmidt Mahmud Aboud ohne vorangegangene Provokation angriff und dass Polizist Gerold Müller zwei Minuten nach Beginn der Auseinandersetzung erschien und Mahmud Aboud den tödlichen Schlag versetzte. Ermittlungen ergaben, dass Polizist Gerold Müller Mitglied von Chatgruppen ist, in denen Kollegen rechtsextremistische Nachrichten austauschen. Die Staatsanwaltschaft erwägt nun, Gerold Müller einer Straftat anzuklagen.

Was ist im eben beschriebenen Szenario passiert? Bitte kreuzen Sie alle zutreffenden Aussagen an.

- Syrischer Flüchtling Mahmud Aboud ist Angeklagter
- Syrischer Flüchtling Mahmud Aboud ist Opfer
- Gerold Müller ist Angeklagter
- Gerold Müller ist Opfer
- Das Opfer ist verstorben
- Das Opfer lebt

Stellen Sie sich vor, ein Richter im Verfahren gegen den Angeklagten Gerold Müller aufgrund des Todes des syrischen Flüchtlings Mahmud Aboud zu sein. In welchen Anklagepunkten sollte Gerold Müller schuldig gesprochen werden?
 Bitte kreuzen Sie alle Punkte an, in denen Gerold Müller Ihrer Meinung nach angeklagt werden soll. Die Beschreibung der Straftaten befindet sich neben den möglichen Anklagepunkten.

 Möglicher Anklagepunkt:

- Keine Straftat (was Gerold Müller gemacht hat mag falsch, aber nicht illegal sein)
- Fahrlässige Körperverletzung mit Todesfolge (Keine vorsätzliche Tötung oder Verletzung)
- Vorsätzliche Körperverletzung mit Todesfolge (Keine vorsätzliche Tötung, aber vorsätzliche Verletzung - nicht unbedingt aufgrund rassistischer Motive)
- Totschlag (vorsätzliche Tötung, ohne niedere Motive, wie Rassismus)
- Mord (Vorsätzliche Tötung, mit niederen Motiven, wie Rassismus)

Im Falle, dass Gerold Müller der fahrlässigen Körperverletzung mit Todesfolge schuldig gesprochen wird, wie hoch sollte die Gefängnisstrafe Ihrer Einschätzung nach sein?

- 1 Monat bis 2 Jahre
- 3 bis 5 Jahre
- 5 bis 7 Jahre
- 8 bis 10 Jahre
- 10 oder mehr Jahre

Im Falle, dass Gerold Müller der vorsätzlichen Körperverletzung mit Todesfolge schuldig gesprochen wird, wie hoch sollte die Gefängnisstrafe Ihrer Einschätzung nach sein?

- 1 Monat bis 2 Jahre
- 3 bis 5 Jahre
- 5 bis 7 Jahre
- 8 bis 10 Jahre
- 10 oder mehr Jahre

Im Falle, dass Gerold Müller des Totschlags schuldig gesprochen wird, wie hoch sollte die Gefängnisstrafe Ihrer Einschätzung nach sein?

- 1 Monat bis 2 Jahre
- 3 bis 5 Jahre
- 5 bis 7 Jahre
- 8 bis 10 Jahre
- 10 oder mehr Jahre

Im Falle, dass Gerold Müller des Mordes schuldig gesprochen wird, wie hoch sollte die Gefängnisstrafe Ihrer Einschätzung nach sein?

- 1 Monat bis 2 Jahre
- 3 bis 5 Jahre
- 5 bis 7 Jahre
- 8 bis 10 Jahre
- 10 oder mehr Jahre

Bedingung 2A:

Gerold Müller hat folgende Stellungnahme an die Familie Abouds abgegeben:
Das mit Ihrem Sohn tut mir leid. Ich wusste nicht, wie viel Aggression Flüchtlinge in ihrem täglichen Leben erfahren müssen. Ich fühle mich so schrecklich. Ich dachte, ich würde helfen, und ich hatte nie die Absicht, ernsthaften Schaden anzurichten. Es tut mir zutiefst leid und ich wünschte, ich könnte meinen Fehler wiedergutmachen. Ich lerne eine Menge und hoffe einfach, dass wir durch diesen schrecklichen Verlust irgendwie lernen können, einander zu akzeptieren.

Bedingung 2B:

Gerold Müller hat folgende Stellungnahme an die Familie Abouds abgegeben:
 Es tut mir leid wegen Ihres Sohnes. Ich wusste nicht, dass er diesmal nicht der Angreifer war. Ich meine, man hört ständig, dass Leute von Leuten wie ihm angegriffen werden, und ich wollte nur helfen, meine Gemeinde zu schützen. Ich weiß nicht, was er im Park gemacht hat, und ich habe ihn nicht einmal sehr fest geschlagen, es war einfach großes Pech, dass er so schwer gestürzt ist, aber ich glaube nicht, dass ich viel Schuld daran habe.

Angenommen, Sie wären Richter im Prozess und würden Gerold Müller einer Straftat schuldig sprechen, zu welchem Urteil würden Sie gelangen?

- Sehr milde Bestrafung (1)
- (2)
- (3)
- (4)
- (5)
- (6)
- Sehr harte Bestrafung (7)

Kreuzen Sie das Urteil an, das Ihnen am angebrachtesten erscheint:

- Freispruch (keine Strafe)
- Geringe Geldstrafe
- Mittlere Geldstrafe
- Hohe Geldstrafe
- Kurzes Gefängnisurteil
- Mittleres Gefängnisurteil
- Langes Gefängnisurteil

Wie sehr stimmen Sie folgenden Aussagen zu?
 Bitte geben Sie an, inwiefern Sie den Aussagen zustimmen bzw. nicht zustimmen.

- In Deutschland lebende Flüchtlinge belasten das Sozialsystem.
- In Deutschland leben zu viele Flüchtlinge.
- Bei der Prüfung von Asylanträgen sollte der Staat großzügig sein.
- Die meisten Flüchtlinge haben keine wirkliche Angst vor Verfolgung in ihren Heimatländern.

Stimme überhaupt nicht zu - Stimme voll und ganz zu (7er Skala)

Wie sehr stimmen Sie folgenden Aussagen zu?
Bitte geben Sie an, inwiefern Sie den Aussagen zustimmen bzw. nicht zustimmen.

- Manche Gruppen von Menschen müssen an ihrem Platz gehalten werden.
- Es ist wahrscheinlich gut, dass bestimmte Gruppen oben stehen und andere unten.
- Eine ideale Gesellschaft erfordert, dass einige Gruppen oben stehen und andere Gruppen unten.
- Manche Gruppen von Menschen sind anderen Gruppen einfach unterlegen.
- Gruppen, die unten sind, sollte genauso viel zustehen wie Gruppen, die oben sind.
- Keine Gruppe sollte in der Gesellschaft dominieren.
- Gruppen, die unten stehen, sollten nicht gezwungen werden, an ihrem Platz zu bleiben.
- Die Dominanz einer bestimmten Gruppe ist ein schlechtes Prinzip.
- Wir sollten nicht auf Gruppengleichheit drängen.
- Wir sollten nicht versuchen zu garantieren, dass jede Gruppe die gleiche Lebensqualität hat
- Es ist ungerecht zu versuchen, Gruppen gleich zu machen.
- Gruppengleichheit sollte nicht unser oberstes Ziel sein.
- Wir sollten daran arbeiten, allen Gruppen die gleichen Chancen zu geben, erfolgreich zu sein.
- Bitte kreuzen Sie hier "stimme eher zu" an. Danke.
- Wir sollten tun, was wir können, um die Bedingungen für verschiedene Gruppen anzugleichen.
- Egal wie viel Aufwand es erfordert, sollten wir uns darum bemühen, dass alle Gruppen die gleichen Chancen im Leben haben.
- Gruppengleichheit sollte unser Ideal sein.

Stimme überhaupt nicht zu - stimme voll und ganz zu (7er Skala)

Bitte machen Sie zum Schluss noch ein paar Angaben über Ihre Person.
Im Folgenden sehen Sie eine Leiter. Ganz oben auf der Leiter stehen die Menschen, denen es am besten geht, die das meiste Geld, die höchste Bildung und die besten Jobs haben. Ganz unten stehen die Menschen, denen es am schlechtesten geht, die das geringste Geld, die geringste Bildung, die schlechtesten Jobs oder gar keine Arbeit haben.
 Bitte markieren Sie die Sprosse, die Ihrer Meinung nach am besten darstellt, wo Sie auf der Leiter stehen.

1 (1)

2 (2)

3 (3)

4 (4)

5 (5)

6 (6)

7 (7)

Bitte geben Sie Ihr Geschlecht an.

- Weiblich
- Männlich
- Anders, und zwar __________________________________________________

Alter

Bitte geben Sie Ihr Alter (in Jahren) an.

________________________________________________________________

Sind Sie in Deutschland geboren?

- Ja
- Nein

Haben Sie die deutsche Staatsangehörigkeit?

- Ja
- Nein

Welcher Religion fühlen Sie sich zugehörig?

- Katholizismus/Christentum
- Judentum
- Islam
- Buddhismus
- Hinduismus
- Keine
- Andere (bitte angeben) __________________________________________________

Welchen höchsten allgemeinbildenden Schulabschluss haben Sie?

- Schüler*in an einer allgemeinbildenden Schule
- Von der Schule abgegangen ohne Schulabschluss
- Hauptschulabschluss, Volksschulabschluss oder vergleichbarer Abschluss
- Realschulabschluss, Mittlere Reife oder vergleichbarer Abschluss
- Fachhochschulreife
- Abitur, Allgemeine oder Fachgebundene Hochschulreife, Gymnasium

Welchen höchsten beruflichen Ausbildungsabschluss haben Sie?

- Noch in Ausbildung, Studium
- Kein beruflicher Abschluss und bin nicht in beruflicher Ausbildung
- Lehre (beruflich-betriebliche Berufsausbildung)
- Berufsqualifizierender Abschluss einer beruflichschulischen Ausbildung
- Abschluss einer Fach-, Meister- Technikerschule, Verwaltungs- und Wirtschaftsakademie, Berufs- oder Fachakademie
- Bachelor, Diplom (FH)
- Master, Diplom (Uni), Magister, Staatsexamen, Promotion

Welche Partei würden Sie wählen, wenn am nächsten Sonntag Bundestagswahl wäre?

- SPD
- CDU/CSU
- Grüne
- FDP
- AfD
- Linke
- Andere: __________________________________________________

In der Politik spricht man häufig von "links" und "rechts". Bitte geben Sie an, wie links oder rechts Sie sich selbst verorten würden.

- Links (0)
- (1)
- (2)
- (3)
- (4)
- (5)
- (6)
- (7)
- (8)
- (9)
- Rechts (10)
